# Control by the brain of vitamin A homeostasis

**DOI:** 10.1016/j.isci.2023.107373

**Published:** 2023-07-13

**Authors:** Peter I. Imoesi, Cristian M. Olarte-Sánchez, Lorenzo Croce, William S. Blaner, Peter J. Morgan, Lora Heisler, Peter McCaffery

**Affiliations:** 1 Institute of Medical Sciences, University of Aberdeen, Foresterhill, Aberdeen AB25 2ZD, Scotland, UK; 2 Rowett Institute, School of Medicine, Medical Sciences and Nutrition, University of Aberdeen, Foresterhill, Aberdeen AB25 2ZD, Scotland, UK; 3 Department of Medicine, Vagelos College of Physicians and Surgeons, Columbia University, 630 West 168th Street, New York, NY 10032, USA

**Keywords:** Natural sciences, Biological sciences, Physiology, Neuroscience, Endocrinology

## Abstract

Vitamin A is a micronutrient essential for vertebrate animals maintained in homeostatic balance in the body; however, little is known about the control of this balance. This study investigated whether the hypothalamus, a key integrative brain region, regulates vitamin A levels in the liver and circulation. Vitamin A in the form of retinol or retinoic acid was stereotactically injected into the 3^rd^ ventricle of the rat brain. Alternatively, retinoids in the mouse hypothalamus were altered through retinol-binding protein 4 (*Rbp4*) gene knockdown. This led to rapid change in the liver proteins controlling vitamin A homeostasis as well as vitamin A itself in liver and the circulation. Prolonged disruption of *Rbp4* in the region of the arcuate nucleus of the mouse hypothalamus altered retinol levels in the liver. This supports the concept that the brain may sense retinoids and influence whole-body vitamin A homeostasis with a possible “vitaminostatic” role.

## Highlights

• Vitamin A is kept at a constant concentration in the blood but it is unknown how
• Hypothalamic tanycytes have the potential to sense vitamin A
• Vitamin A application to the hypothalamus alters systemic vitamin A distribution
• This reveals a possible role for the brain in the control of vitamin A homeostasis

## Graphical abstract

**Figure:**
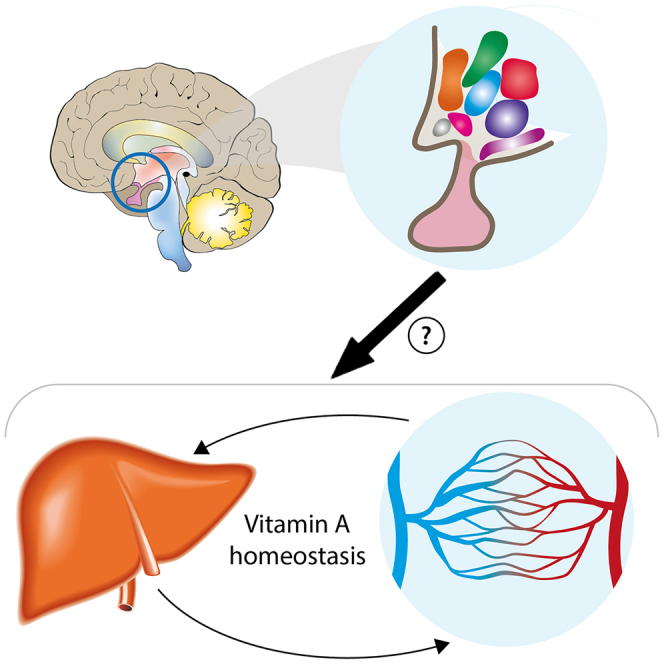

## Introduction

Vitamin A is essential for growth, immune function, the visual system, and reproduction^1^ and is unusual amongst vitamins as it is extensively stored in the body.^2^ This storage means, despite peaks and troughs of dietary vitamin A intake, a constant concentration of vitamin A in the form of retinol (ROL) can be maintained in blood. This ROL concentration of 1–3 μM (depending on species) maintains the uninterrupted supply of vitamin A to all cells.^3^ This is preserved even in cases of vitamin A dietary deficiency or excess intake^4^ and only disrupted when deficiency is chronic, resulting in a sharp fall of circulating plasma ROL. This system thus ensures constant delivery of ROL to tissues in which this substrate is oxidized to retinoic acid (RA) which acts and is then rapidly degraded, and RA levels in the body and plasma of this active metabolite are kept low, around 7 nM.^5^ A fundamental unresolved question is how the vitamin A concentration in the circulation is maintained.

The liver is the major storage organ for vitamin A. After the ingestion of vitamin A in the diet, the liver takes up ROL for either storage or immediate delivery to tissues around the body, bound to retinol-binding protein 4 (RBP4) and transthyretin.^6^^,^^7^ A simple feedback mechanism has been proposed to regulate vitamin A homeostasis involving RA, as the metabolite of vitamin A, promoting vitamin A storage.^8^ However, there is evidence for extra-hepatic control^9^ and we postulate that the hypothalamus is part of this mechanism.

The hypothalamus regulates physiological balance including temperature, food intake, water load, energy metabolism, stress levels, and temporal rhythms, as well as regulating the timing of reproduction and sexual maturity.^10^^,^^11^ Input from the environment, essential for the hypothalamus to sustain appropriate physiological balance, comes from sensory, autonomic, and neuro-humoral inputs, and the hypothalamus contains its own sensors to register internal changes in blood and cerebrospinal fluid (CSF). Putative sensors for ROL exist in the hypothalamus via tanycytes^12^^,^^13^ which make up a blood-CSF barrier in the hypothalamus receiving signals from both blood and CSF. Tanycytes are amongst the few cells in the brain able to convert ROL to RA^14^ which activates specific retinoic acid receptors (RARs). The proteins that capture ROL into the cell (the ROL transporter RBP4 and ROL receptor Stra6) are both enriched in tanycytes^15^ and so would provide a signaling system that responds to ROL.

The potential for the hypothalamus, or even the brain more broadly, to control whole-body vitamin A homeostasis has not been reported. As a test of this hypothesis ROL or RA levels were raised in the hypothalamus by stereotactic injection into the rat 3^rd^ ventricle adjacent to the tanycytes of the hypothalamus. This would mimic the effect of high levels of ROL in the CSF or circulation. ROL, RA, and storage retinyl esters, were measured in the blood, liver, and other vitamin A storage organs. As a result of retinoid injection into the 3^rd^ ventricle, vitamin A homeostatic genes and proteins were found to change within 6 h in the liver, but not other storage organs. Further, the induction of longer-term changes in retinoid balance in the arcuate nucleus of the mouse hypothalamus via *Rbp4* knockdown led to significant changes in liver ROL levels. This supports the hypothesis of a regulatory role for the hypothalamus on whole-body vitamin A homeostasis.

## Results

### Enzymes converting retinol to retinoic acid in hypothalamic tanycytes

**Figure 1:**
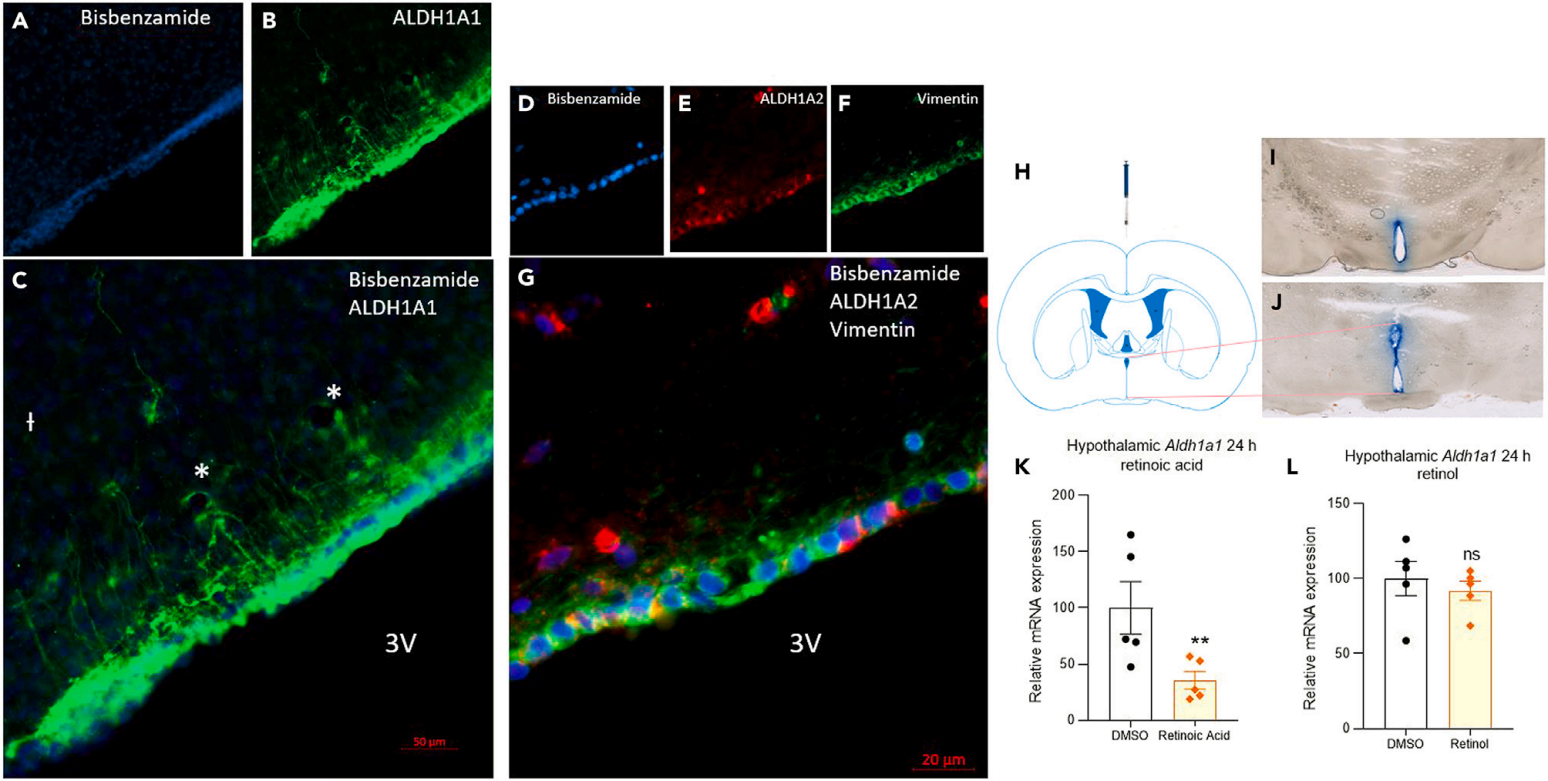
ALDH1A1 and ALDH1A2 enzymes converting ROL to RA in hypothalamic tanycytes and quantitation of *Aldh1A1* transcript in the hypothalamus after RA or ROL injection (A–L) ALDH1A1 is present in tanycytes and around blood vessels (marked with ∗) in the rat in the vicinity of the arcuate nucleus with ALDH1A1 transported along tanycytic processes (A-C). In the human hypothalamus cells lining the third ventricle with nuclei labeled with bisbenzamide (D) express vimentin in their cytoplasm with expression in processes implying these are tancytes (F and G) and these cells are also positive for ALDH1A2 (E, G). The tanycytic processes are substantially shorter in the human (G) than the rat (C). Scale bar (C) 50 μM, (G) 20 μM. Injection of Evans blue dye into the third ventricle (H) reaches the cells lining the ventricle along the rostral (I) and caudal (J) length of the hypothalamus. RA results in a reduction in hypothalamic *Aldh1a1* in 24-h (K) but there is no significant change in the hypothalamus in this transcript following hypothalamic injection of ROL (L). n = 5, data are represented as mean ± SEM (unpaired Student’s t-Test statistical test applied with ∗∗p ≤ 0.01).

Tanycytes are specialized ependymal cells lining the 3^rd^ ventricle that can sense nutrients and metabolic hormones in the blood and CSF. In the rodent, tanycytes send long processes into the hypothalamus to communicate with hypothalamic cells.^16^ We have previously shown in the rodent that tanycytes are amongst the few cells in the brain that express the ALDH1A1 enzymes (also known as RALDH1) necessary to convert ROL to RA.^17^ The extensive processes of tanycytes contain the ALDH1A1 enzyme, for example in the rat (Figures 1A–1C), generating RA from ROL and allowing it to transmit a signal via retinoic acid receptors (RARs) present in the hypothalamus.^14^^,^^18^^,^^19^ We show that human tanycytes also express RA synthesizing enzymes, in this case ALDH1A2 (also known as RALDH2) (Figures 1D–1G), reflecting that different RALDHs are expressed in the tanycytes of different species.^14^ Thus, tanycytes could act as sensors for ROL by converting it to bioactive RA.

We next tested whether the application of ROL and its RA metabolite to the region of the tanycytes will trigger changes in body vitamin A storage. For these tests ROL and RA were injected into the 3^rd^ ventricle of the rat brain exposing the tanycytes to high levels of these retinoids. Preliminary injection with dye showed such injected compound reach the cells lining the third ventricle (Figures 1H–1J). The experimental group was injected with 5 μL of 10^−4^ M all-*trans* RA or all-*trans* ROL in 10% DMSO in saline and the control group with 5 μL 10% DMSO in saline. This amount of RA or ROL would give an approximately 5.5 μM concentration in the 90 μL volume of the CSF^20^ with the constant flow of 2 μL/min this concentration though will become quickly diluted. Although the injected ROL or RA is unlikely to move from the compartment of the brain to the rest of the body if it was averaged across the body, it would only reach approximately 1.7 nM, similar to normal circulating levels of RA^21^ and far below the normal 1–3 μM levels of ROL. Twenty-four hours after RA treatment (Figure 1K), there was a local change in gene expression in the hypothalamus in the form of a compensatory downregulation of the RA synthesizing enzyme ALDH1A1. This was not evident 24 h following the injection of ROL (Figure 1L).

### Effect of 3^rd^ ventricle retinol or retinoic acid injection on liver retinoid-related transcript expression

**Figure 2:**
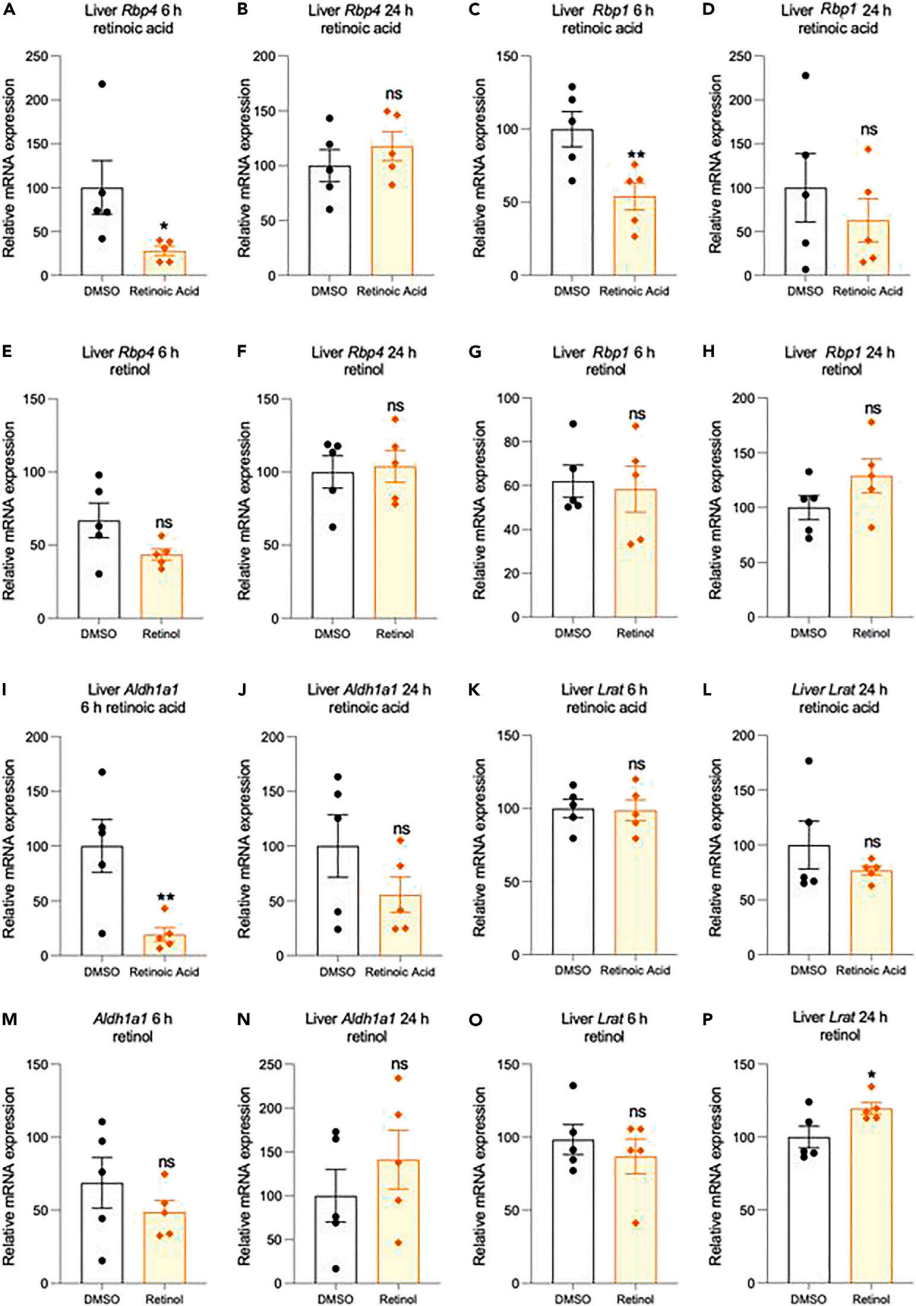
Effect of RA or ROL injection into the 3^rd^ ventricle on *Rbp4*, *Rbp1*, *Aldh1a1*, and *Lrat* transcript expression in the liver (A–P) Injection of RA into the 3^rd^ ventricle induces a significant reduction in both binding protein transcripts *Rbp4 and Rbp1* in the liver 6-h after exposure (A, C) but has no significant effect after 24-h (B, D). In comparison ROL had no action on either *Rbp4 and Rbp1* at 6- or 24-h (E, F, G, H). For the two transcripts encoding enzymes, *Aldh1a1* and *Lrat*, injection of RA into the 3^rd^ ventricle induces a significant reduction in liver *Aldh1a1* after 6- but not 24-h (I, J) but has no significant action on *Lrat* at either time (K, L). ROL had no significant action on either *Aldh1a1* or *Lrat* (M, N, O) except inducing a small increase at 24-h (P). RA n=5; ROL experimental and control n = 5 & 9, data are represented as mean ± SEM (unpaired Student’s t-Test statistical test applied with ∗p ≤ 0.05, ∗∗p ≤ 0.01).

To test whether these induced changes in hypothalamic retinoid levels may then alter whole-body vitamin A homeostasis, alteration in genes controlling this system in the central vitamin A storage organ of the liver was determined. Just 6-h after RA injection in the rat 3^rd^ ventricle, decreases in the expression of transcripts of retinoid binding proteins in the liver were evident. Specifically, 3^rd^ ventricle RA reduced retinol-binding protein 4 (RBP4) transcript, which is synthesised by hepatocytes to carry ROL outside of the liver (*Rbp4*, Figure 2A) and cellular-retinol binding protein (*Rbp1*, Figure 2C), which carries ROL in liver cells. Although a similar decline was evident with ROL treatment this was to a lesser extent and not significant (Figures 2E and 2G). These changes were no longer evident by 24-h for either RA or ROL treatment of the brain (Figures 2B, 2D, 2F, and 2H).

Genes involved in retinoid metabolism in the liver were next examined following the injection of retinoids in the 3^rd^ ventricle. Liver transcript of the enzyme ALDH1A1, catalyzing the last step of the conversion of ROL to RA, and potentially on to further catabolism, significantly declined in expression with 3^rd^ ventricle RA treatment at 6-h and non-significant at 24-h (*Aldh1a1* Figures 2I and 2J). There was no significant effect with 3^rd^ ventricle ROL infusion on *Aldh1a1* (Figures 2M and 2N). Transcripts of lecithin-retinol acyltransferase (*Lrat*) were also measured, encoding the enzyme esterifying ROL to retinyl ester for storage but this did not change with ROL or RA injection into the 3^rd^ ventricle (*Lrat* Figures 2K, 2L, and 2O) except for a small significant increase at 24-h with ROL treatment (Figure 2P). The results indicated that only the bioactive product of vitamin A, RA, injected into the brain induced large and significant changes in the liver transcripts controlling retinoid homeostasis.

### Effect of 3^rd^ ventricle retinoic acid injection on liver retinoid-related protein expression

**Figure 3:**
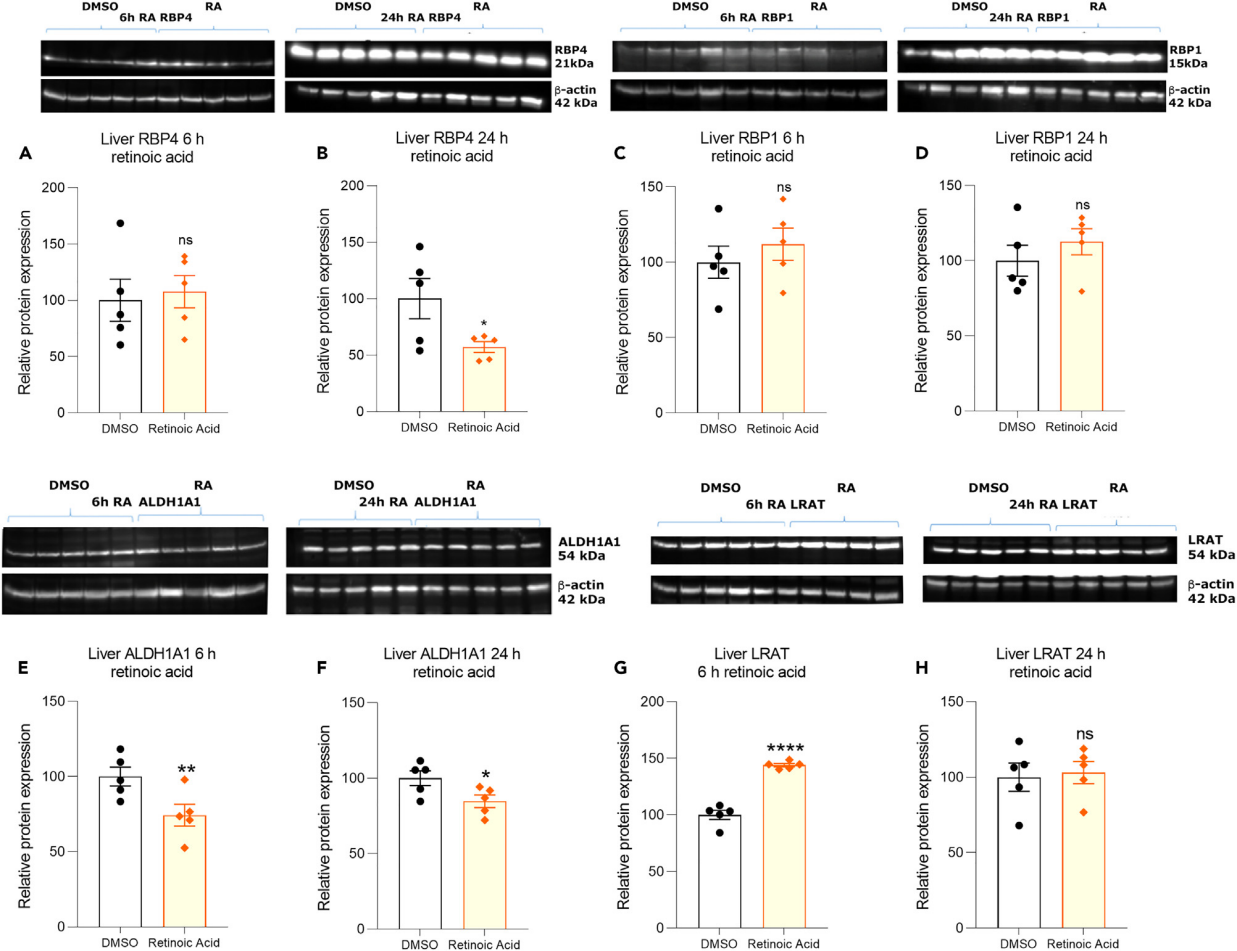
Effect of RA injection into the 3^rd^ ventricle on RBP4, RBP1, ALDH1A1 and LRAT protein expression in the liver (A–H) Injection of RA into the 3^rd^ ventricle induces a significant reduction in liver binding protein RBP4 at 24, but not 6-h (B, A) but has no significant action on RBP1 expression (C, D). For the enzymes quantified, injection of RA into the 3^rd^ ventricle induces a significant reduction in liver ALDH1A1 expression at both 6 and 24-h (E, F) and also induces a significant increase in LRAT at 6, but not 24-h (G, H) n=5, data are represented as mean ± SEM (unpaired Student’s t-Test statistical test applied with ∗p ≤ 0.05, ∗∗p ≤ 0.01, ∗∗∗∗p ≤ 0.0001).

The influence of brain RA infusion on the protein products of such retinoid metabolic genes in the liver was then examined. This produced significant reductions in liver RBP4 at 24-h, but not at 6-h (Figures 3A and 3B). Brain infusion with RA produced no significant change in liver RBP1 (Figures 3C and 3D). Investigating the changes in liver ALDH1A1 after 3^rd^ ventricle RA infusion revealed that this retinoid metabolic enzyme fell significantly by 6-h and a reduction was maintained at 24-h (Figures 3E and 3F). Protein levels however of liver LRAT significantly increased by 6-h following RA application to the 3^rd^ ventricle (Figure 3G), unlike the corresponding transcript which remained unchanged (Figure 2K). Liver LRAT protein levels were restored to control levels by 24-h (Figure 3H). These data indicate strong effects of RA applied to the 3^rd^ ventricle on the capacity of the liver to process and transport vitamin A.

### Effect of 3^rd^ ventricle retinoic acid injection on retinoid levels in the body, and absence of effect on kidney and lung

**Figure 4:**
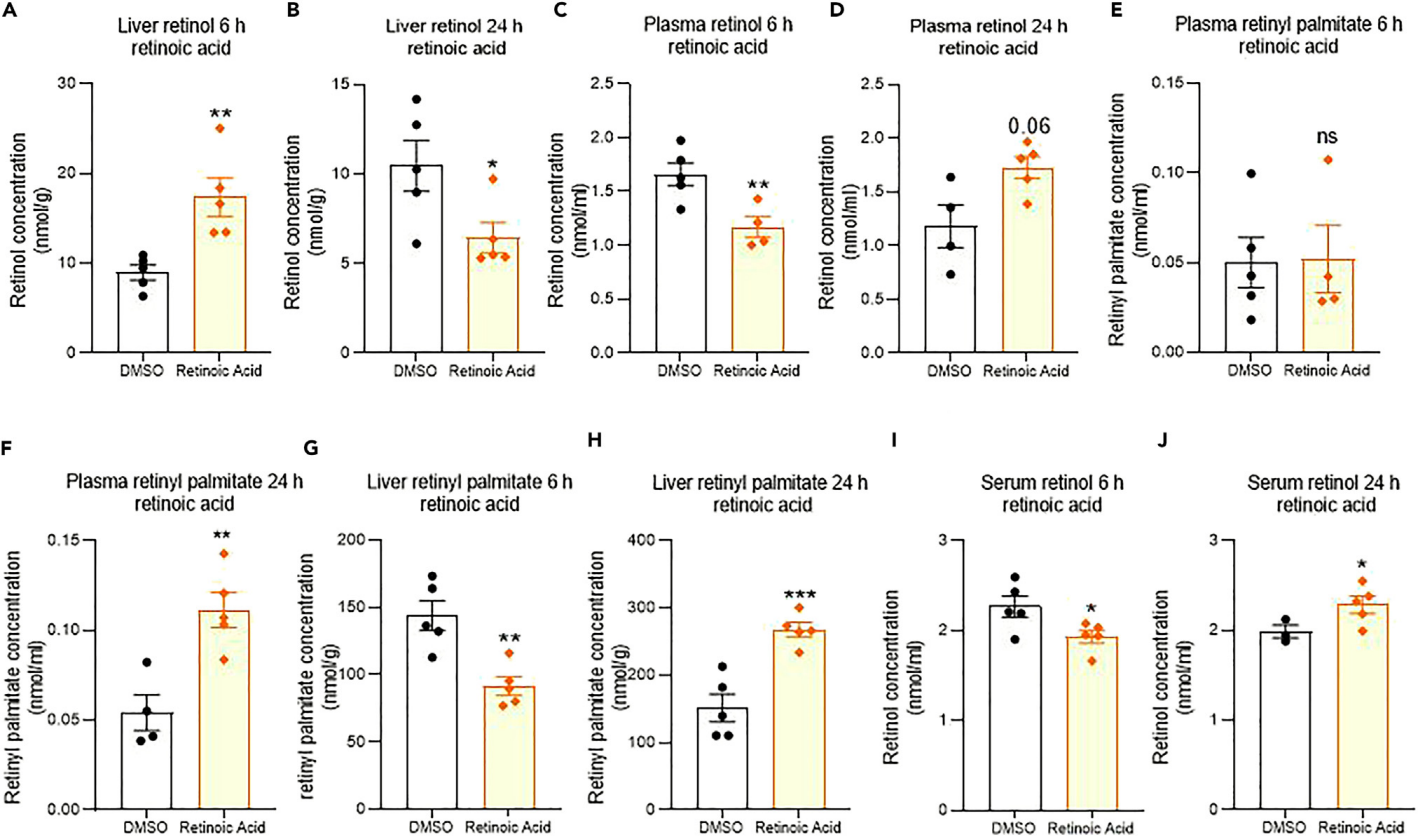
Effect of RA injection into the rat 3^rd^ ventricle on retinoid levels in the body (A–J) Injection of RA into the hypothalamus induces a significant increase in liver ROL at 6-h and then decreasing significantly below control by 24-h (A, B). Plasma and serum ROL contrasts with the inverse pattern (C, D, I, J) significantly falling below control at 6 and then rising at 24-h (although the change in plasma is not significant at 24-h, D). Plasma retinyl esters are unchanged at 6-h but increased at 24-h (E, F). A similar inverse pattern to liver ROL is evident in liver retinyl esters, significantly falling below control at 6-h and then rising to double that of control by 24-h (G, H). A–J n=5, data are represented as mean ± SEM (unpaired Student’s t-Test statistical test applied with ∗p ≤ 0.05, ∗∗p ≤ 0.01, ∗∗∗p ≤ 0.001).

**Figure 5:**
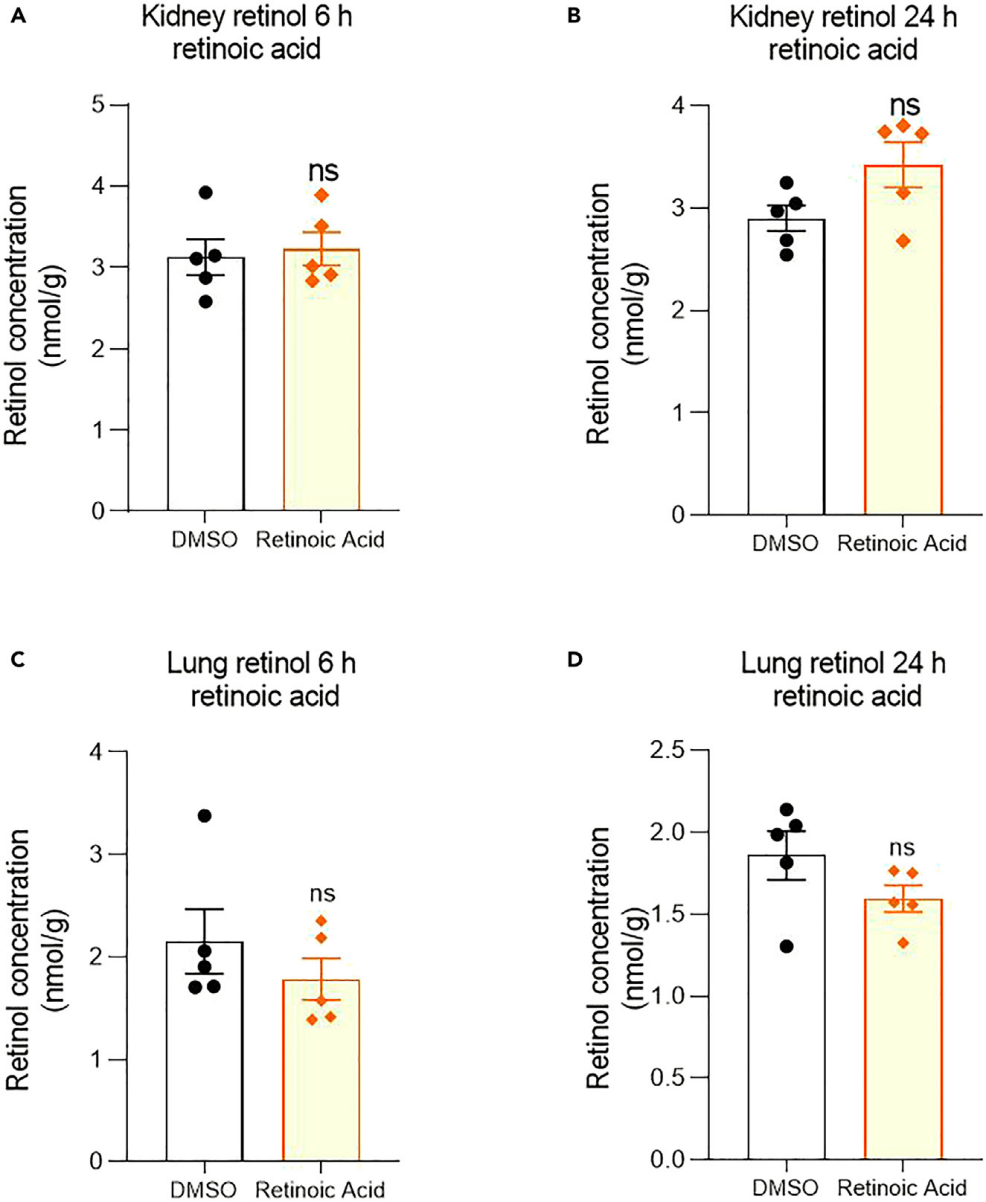
Effect of RA injection into the hypothalamus on ROL and retinyl palmitate levels in the kidney and lung (A–D) Injection of RA into the hypothalamus results in no significant changes in ROL in either the kidney at 6 and 24-h (A, B) nor in the lung (C, D). A-D n = 5, data are represented as mean ± SEM.

We next determined whether there were concomitant changes in the retinoids these proteins controlled. The resulting change in liver ROL and retinyl palmitate were examined after the injection of RA in the 3^rd^ ventricle of the rat brain. Six hours after brain RA injection, ROL levels significantly rose in the liver but by 24-h had decreased compared to controls (Figures 4A and 4B). This was mirrored by changes in retinyl esters which significantly fell at 6-h but were above controls by 24-h (Figures 4G and 4H). The effects on ROL blood levels were investigated given this is the route of ROL delivery to the body, measuring in both plasma and serum. Both preparations showed changes inverse to those in the liver which may be explained by the movement of ROL from plasma to liver - with a ROL decline in plasma and serum at 6-h and rise at 24-h (Figures 4C, 4D, 4I, 4J). This change however was not significant in plasma at 24-h (Figure 4D). Retinyl palmitate was also measured in the plasma, and this was unchanged at 6-h but increased by 24-h (Figures 4E and 4F). Finally, it was examined whether the injections of RA in the 3^rd^ ventricle influenced secondary vitamin A storage areas such as the kidney and lung. There was no significant change in the ROL levels in either of these two organs at 6- or 24-h post-injection (Figures 5A–5D).

### Effect of *Rbp4* knockdown in the hypothalamus on retinoid levels in the body

**Figure 6:**
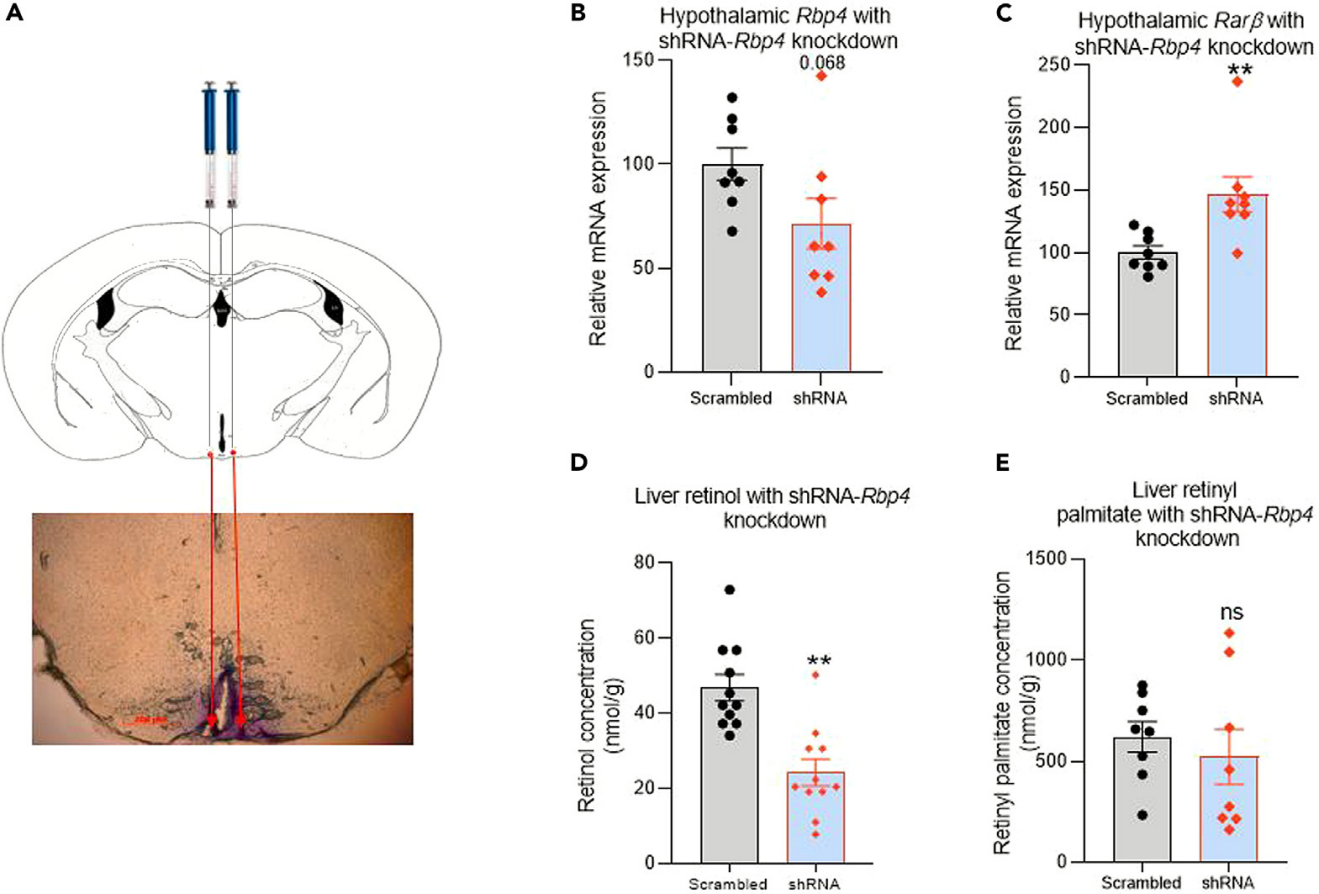
Effect of *Rbp4* knockdown in the mouse hypothalamus on retinoid levels in the body (A–E) Injection of cresyl violet to reach the region of the arcuate nucleus was confirmed (A) before the injection of shRNA designed to knockdown *Rbp4* in the area of the arcuate nucleus. shRNA knockdown of *Rbp4* did not reach significance (B, p < 0.068) but this knockdown was sufficient to raise transcript of *Rarb* (C), a transcript with sensitive RA response element and implying a local increase in RA levels. Knockdown of hypothalamic *Rbp4* resulted in a significant decrease in liver ROL (D) although there was no change in retinyl palmitate (E). A–E n=5, B-E n=9, data are represented as mean ± SEM (unpaired Student’s t-Test statistical test applied with ∗∗p ≤ 0.01).

The longer-term effects of the disruption of retinoid levels in the hypothalamus were also explored to investigate whether this interfered with whole-body vitamin A homeostasis. This was set up for the mouse brain and disruption of local retinoid signaling in the hypothalamus was achieved by changing the balance of retinol-binding protein 4 (RBP4) in the arcuate nucleus of the hypothalamus. This binding protein is expressed in a variety of neuronal types in the brain including striatum and hippocampus and excitatory neurons of the cortex.^22^ In the mouse hypothalamus, RBP4 is enriched both in agouti-related peptide (AgRP) neurons^23^ and tanycytes.^15^ RBP4 binds ROL and also RA and retinyl esters with similar affinity^24^ and knockdown will increase available retinoids for signaling. Following the local injection of short-hairpin adeno-associated virus (shRNA AAV) vector into the mouse arcuate nucleus (Figure 6A), a decline of *Rbp4* transcript 4-week was evident when the entire hypothalamus was quantified, but this was not significant (Figure 6B). However, this was sufficient to increase RA signaling in the area as indicated by the measurement of induction of *Rar β* (Figure 6C) as a reporter of RA signaling with its highly sensitive retinoic acid response element in its promoter.^25^ This treatment led to a significant decrease in liver ROL (Figure 6D) which parallels the decline in rat liver ROL after 24-h of RA infusion into the rat 3^rd^ ventricle (Figure 4B). In contrast to rat liver retinyl palmitate which increased at 24-h (Figure 4H), retinyl palmitate in the mouse liver did not change significantly (Figure 6E). Taken together, these studies suggest that a system regulating body stores of vitamin A may also exist in the mouse, analogous to that of the rat.

## Discussion

This study is the first evidence that the brain can sense retinoids, and through this, regulate whole-body retinoid balance. This was evaluated in rat with a short-term pulse of retinoid into the 3^rd^ ventricle adjacent to the tanycytes of the hypothalamus, putative sensor cells for these retinoids. This was associated with a rapid (within 6-h) and profound effects on ROL and retinyl ester storage in the liver as well as circulating ROL. A potential driver of this were changes in liver LRAT protein, key in terms of ROL esterification as 80–90% of total retinoids in the body are stored as esters, with retinyl palmitate making up 70–80% of this.^26^ A strong response was found from the injection of RA into the 3^rd^ ventricle, but much weaker for ROL. It was hypothesized that ROL would send a signal in tanycytes by conversion to bioactive RA, and if this is the case it is unsurprising that RA was stronger than ROL. The method of simple injection of retinoid into the 3^rd^ ventricle and its rapid dilution due to CSF flow may not provide the necessary time for sufficient conversion of ROL to RA. This would require future studies perfusing ROL into the 3^rd^ ventricle. A test of the hypothesis was also performed in the mouse taking a different approach; rather than injecting retinoids into the 3^rd^ ventricle adjacent to the rat hypothalamus, a viral vector carrying shRNA for knockdown of *Rbp4* was injected in the vicinity of the mouse arcuate nucleus. It was presumed this will induce *Rar β* which implied an increase in RA levels in this region^27^ over a longer period than a pulse of retinoid. This treatment was accompanied by decreased ROL in the mouse liver which parallels the changes in rat liver after 24-h. These findings suggest that the mouse also has a system by which the hypothalamus can communicate with the liver to control ROL levels.

RA injection into the 3^rd^ ventricle of the rat resulted in significant changes in vitamin A homeostasis in the liver as determined by altered expression of genes encoding proteins controlling retinoid metabolism. The gene transcripts for binding proteins *Rbp1* and *Rbp4* and enzyme *Aldh1a1* changed rapidly in expression in the liver with RA injection, showing strong decline after 6-h, a response lost by 24-h. In contrast transcript for *Lrat* remained unresponsive to RA or ROL. These changes in genes of the vitamin A homeostatic system imply that the hypothalamus communicates with the liver when stimulated by retinoids. The route by which this communication takes place was not determined in this study and may involve hormonal or nervous control.^28^ The route may have parallels to the mechanism by which the hypothalamus regulates glucose homeostasis via the vagus nerve.^29^

Given that only the RA treatment of the 3^rd^ ventricle was associated with significant changes in liver transcript, this was the focus to study corresponding changes in protein. Although the *Rbp1* transcript declined with the RA treatment of the 3^rd^ ventricle, its protein levels were unchanged, up to 24-h, suggesting that the protein is stable over this period. RBP4 expression however corresponded with transcript, significantly declining although with a lag phase from transcript declining only after 24-h. Expression of the ALDH1A1 enzyme also reflected the change in transcript declining in expression by 6-h and remaining at low levels by 24-h. LRAT protein, in contrast, differed markedly from transcript. Whereas liver transcript remained unchanged with RA injection into the 3^rd^ ventricle, the protein climbed significantly at 6-h, suggesting that the retinoid-induced signal in the hypothalamus may independently regulate both transcript and protein levels in the liver to control homeostasis. LRAT is the only enzyme that catalyses the transesterification of ROL in liver for storage^30^ and subsequent formation of lipid droplets drives the movement of ROL into the liver.^31^ Induction of LRAT levels in the liver by high amounts of vitamin A would remove ROL from the blood to storage sites in the liver.

The levels of retinoids in the liver and plasma were measured, with the hypothesis that RA injection into the 3^rd^ ventricle will mimic high vitamin A levels to promote the movement of ROL into the liver and reduce circulating levels. This was observed with levels of ROL significantly falling after 6-h in blood and levels in the liver rising. The mechanism for this early increase of ROL into the liver however is unknown and paralleled a decrease in retinyl palmitate. The expected increase in retinyl palmitate resulting from the movement of ROL into the liver and the measured increase in LRAT at 6 h not occurring until 24-h, implying a lag phase in the generation of the storage ester. By this 24-h period the generation of retinyl ester may have led to lowered liver levels of ROL. Interestingly, also by 24-h, levels of ROL in the blood increased relative to 6 h, implying that the pulse of RA into the 3rd ventricle had only a transient effect on vitamin A homeostasis.

Vitamin A is essential for normal function and reproduction. A fundamental and unresolved question is how vitamin A levels in the body are regulated and maintained. This study provides the first evidence that the brain senses retinoids and regulates body vitamin A via actions on the liver and revealing a possible new mechanism for the regulation of vitamin A homeostasis.

### Limitations of the study

This study shows that alterations in retinoid concentrations local to the hypothalamus result in large changes in the systems controlling vitamin A homeostasis in the liver and circulation, but not other organs. The mechanism by which the hypothalamus communicates with the liver however is unknown and may be nervous or hormonal. The study investigated just two time points and significant changes were evident even at the shortest (6-h) time point indicating that the mechanism is rapid, and analysis of earlier time points will be necessary to determine how rapid. The study found that RA, the active metabolite of vitamin A, showed the largest effect while the effect of ROL was weaker. Although it is hypothesized that ROL will act via its RA metabolite, ROL will be expected to have the same effect over a prolonged period and a complete test of our hypothesis will require ROL treatment in the hypothalamus over a sustained time. That a sustained shift in ROL will alter vitamin A homeostasis is suggested by the influence in the mouse of knockdown of the ROL binding protein encoded by *Rbp4*. It was noted though that a different response was evident in the mouse in that retinyl ester levels did not change, whereas an increase was evident in the rat. Given the different approach to raise levels of RA in the hypothalamus though, it cannot be determined whether this is a species difference.

## STAR★Methods

### Key resources table

**Table:** 

REAGENT or RESOURCE	SOURCE	IDENTIFIER
**Antibodies**		

ALDH1A1 Polyclonal antibody	Proteintech	Cat# 15910-1-AP; RRID: AB_2305276
CoraLite488 conjugated Affinipure Goat Anti-Rabbit IgG (H+L)	Proteintech	Cat# SA00013-2; RRID: AB_2797132
Purified anti-Vimentin	BioLegend	Cat# 677802; RRID: AB_2565982
Donkey Anti-Mouse IgG H&L (Alexa Fluor® 488)	Abcam	Cat# ab150105; RRID: AB_2732856
Anti-Raldh2 (ALDH1A2)	Merck Millipore	Cat# ABN420
Donkey Anti-Rabbit IgG(H+L) Antibody CF594 Conjugated	Biotium	Cat#201521-1; RRID: AB_10853603
Recombinant Anti-RBP4 antibody [EPR18020-115]	Abcam	Cat# ab188230; RRID: AB_2910554
CRBP I (B-8)	Santa Cruz Biotechnology	Cat# sc-271208; RRID: AB_10610075
ALDH1A1 antibody – Neuronal Marker	Abcam	Cat# ab24343; RRID: AB_2224007
LRAT antibody	Proteintech	Cat# 12815-1-AP; RRID: AB_2297180

**Bacterial and virus strains**		

AAV9-GFP-U6-m-RBP4-shRNA	Vector Biolabs	Lot# 171120#32
AAV9-GFP-U6-scrmb-shRNA	Vector Biolabs	Lot# 170814-171201

**Biological samples**		

Human hypothalamus brain	Professor Colin Smith and Edinburgh Brain and Tissue Bank	N/A

**Chemicals, peptides, and recombinant proteins**		

Isoflurane	IsoFlo®, Zoetis	N/A
Rimadyl® Small Animal Solution	Rimadyl™, Zoetis	N/A
Metacam®	Boehringer Ingelheim, Germany	N/A
All-trans-Retinoic Acid	Sigma-Aldrich	Cas# 302-79-4; SKU: R2625-100MG
Retinol	Sigma-Aldrich	Cas# 68-26-8; SKU: R7632-100MG
Acetonitrile	Fisher Scientific	Cas# 75-05-8
Methanol	Fisher Scientific	Cas# 67-56-1
Dichloromethane	Fisher Scientific	Cas# 75-09-2
SensiMix™ SYBR® Green	Bioline	Cat# QT650-05

**Critical commercial assays**		

RNeasy Mini Kit	Qiagen	Cat# 74104
DNase Solution	Qiagen	Cat# 79254
qScript™ cDNA Synthesis Kit	Quanta Biosciences™	Cat# 95047-100
The Pierce Bicinchoninic Acid (BCA) Kit	ThermoFisher Scientific	Cat# 23235
Enhanced Chemiluminescence Kit	Fisher Scientific	Cat# 11546345

**Experimental models: Organisms/strains**		

Female Mouse: C57BL/6	Medical Research Facility, University of Aberdeen	N/A
Rat: Sprague Dawley Male Rats	Envigo RMS, Inc	N/A

**Oligonucleotides**		

qPCR Primers see Table S1	This study	N/A

**Software and algorithms**		

Roche LightCycler 480 v 1.5 software	Roche LightCycler 480 Instrument	RRID: SCR_020502
SoftMax Pro software v 5.2.	Molecular Devices	RRID: SCR_014240
ImageJ software v 1.52e	NIH Image to Image	RRID: SCR_003070
IBM SPSS Statistic v 25	SPSS Inc.	RRID: SCR_002865
GraphPad Prism v 9.4.1	Prism, GraphPad Software, San Diego, CA	RRID: SCR_002798

**Other**		

Nitrocellulose membrane	GE Healthcare	Cat# 10600008
MyECL Imager	Thermo Scientific	N/A

### Resource availability

#### Lead contact

Information and requests for resources and regents should be directed to the lead contact, Peter McCaffery (peter.mccaffery@abdn.ac.uk).

#### Material availability

This study did not generate new unique reagents.

### Experimental model and study participant details

#### Animals and tissues

15 adult male 8-week-old Sprague Dawley rats (Envigo RMS, Inc.) weighing between 250 – 400 g were used for retinoid injection studies. For *Rbp4* hypothalamic knockdown studies, 18 C57BL/6 adult female mice approximately 10 – 12 weeks old and weighing 20 – 25 g were obtained from the breeding wing of the Medical Research Facility – University of Aberdeen. After a week of acclimatisation, surgeries were performed on the rats or mice respectively using methods described below.^32^^,^^33^^,^^34^ Rats or mice were group housed with access to food (CRM (P) Rat and Mouse Breeder and Grower, standard pelleted diet, Special Diet Services) and water *ad libitum*. All experimental animals were approved by the ethics committee of the University of Aberdeen, in accordance with the UK Home Office guidelines, licensed under the Animals (Scientific Procedures) Act, 1986.

### Methods details

#### Immunohistochemistry

Rat brains for immunohistochemistry were prepared by transcardial perfusion fixation under deep terminal anaesthesia and overnight post-fixation in 4% paraformaldehyde. Tissue was sectioned coronally at 40 μm thickness on a freezing sliding microtome and free-floating tissue was then stored in cryoprotectant mix (30% ethylene glycol, 30% sucrose, 1% polyvinylpyrrolidone in sodium phosphate buffer) in a 12-well plate. Immunohistochemistry was performed by submerging the free-floating sections into newly prepared wells of a 12-well plate containing the required solution. The staining procedure consisted of two washes in PBS lasting 15 minutes each, 2-hours in the required animal’s serum blocking solution (dependent on which secondary antibody was subsequently used, consisting of 10% serum, 0.3% Triton X-100 in PBS), primary antibody diluted in the same blocking solution overnight at 4°C, two 15 minutes PBS washes, secondary antibody diluted in the same blocking solution for 2-hours, 2 × 15 minutes PBS washes, and finally mounting sections onto a microscope slide with cover slips and mounting medium containing 4% paraformaldehyde in 0.1 M sodium phosphate buffer with 2.5% 1,4-Diazabicyclo[2.2.2]octane (DABCO) and 0.004% bisbenazmide to label nuclei. Antibodies used were ALDH1A1 (aldehyde dehydrogenase 1A1/RALDH1, a retinoic acid synthesising enzyme, Proteintech) primary antibody and rabbit-specific CoraLite488 secondary antibodies (Proteintech).^17^

#### Human tissue studies

Paraffin sections through the human hypothalamus were by courtesy of Prof Colin Smith and the Edinburgh Brain and Tissue Bank. To remove paraffin the tissue slides were washed in a glass coplin-jar for 10 minutes in Histo-Clear (National Diagnostics) twice, 5 minutes in 100% ethanol twice, 5 minutes in 95% ethanol, 5 minutes in 70% ethanol, 5 minutes in 50% ethanol twice and finally 5 minutes in PBS twice. For antigen retrieval the slides were first placed in a citrate buffer solution (10 mM sodium citrate with 0.05% Tween® 20, pH 6) in a new coplin-jar covered with polyethylene film with a small (3 – 4 cm) cut to allow a measured amount of evaporation. The citrate buffer jar was then microwaved four times for 5 minutes each, making sure that the buffer was boiling hot after the second heating cycle and topped up with hot water after each cycle, before letting the slides cool down while still submerged for 25 minutes. The slides, after encircling the tissue using a PAP hydrophobic marker to avoid spillages, underwent immunohistochemical staining consisting of a wash in PBS 3 × 5 minutes each, 1 hour in serum blocking solution (as described above) in a dark humidity box, overnight incubation with primary antibody diluted in the same blocking solution – also in a humidity box, 3 × 5 minutes PBS wash, two hours incubation with secondary antibody in the same blocking solution, 3 × 5 minutes PBS wash and finally slides were mounted with cover slips with the above-described mounting medium. Procedure was repeated before mounting for double staining. Antibodies used were ALDH1A2 (aldehyde dehydrogenase 1A2/RALDH2, a retinoic acid synthesising enzyme, Merck Millipore) and vimentin (Biolegend) primary antibody, together with rabbit (Biotium, Alexa Fluor 594) and mouse-specific (Abcam, Alexa Fluor 488) secondary antibodies, respectively.^35^

#### Intraventricular injection of retinoids

The rats were divided into experimental and control groups. Before the start of surgery, anaesthesia was induced with isoflurane (IsoFlo®, Zoetis) in 2% oxygen and the surgical area was clipped with a trimmer (Contura-Wella Professionals) before placing the animal in the stereotactic apparatus (David Kopf Instruments, Tujunga, CA, Model 900) with the incisor bar set at 3.3 mm and both ear bars fixed. Once the animal was on the apparatus with the heating pad (Beurer, UK) in place, the surgical area was disinfected with povidine-iodine antiseptic solution (XHG012, Animalcare) and the eyes were creamed with Viscotears® liquid gel to avoid dryness during surgery. 5 mg/kg of Rimadyl® Small Animal Solution (Rimadyl™, Zoetis) was injected subcutaneously before surgery. Similarly, 1.25 mL of warm saline was injected subcutaneously into both sides of the animal at the start and the end of surgery to avoid dehydration. At the start of the surgical procedure, isoflurane was regulated between 1 – 5% in oxygen. Subsequently, a small incision was made exposing the skull and Marcaine (1:1; v/v) was applied to the incision area to stop initial bleeding. The incision area was then cleaned thoroughly with saline and hydrogen peroxide for clearer exposure of bregma. Following the identification of bregma, the Hamilton Microliter™ (#701) syringe was set on bregma as a reference point for stereotactic coordinates. The following coordinates were set to reach the 3rd ventricle; anteroposterior (AP) – 0.8 mm, mediolateral (ML) – 0.0 mm and the dorsoventral (DV) – 6.5 mm.^36^ A small hole was drilled through the skull. The experimental group (n = 5) were injected with 5 μL of 10^-4^M all-*trans* RA or 5 μL of 10^-4^M all-*trans* retinol (Cat. No. R2625 or R7632, Sigma Aldrich) and the control group (n = 5) were injected with 5 μL of 1% DMSO in 0.9% saline. All drugs were slowly injected at a rate of 0.5 μL every 30 seconds for a period of 5 minutes.

After the injections, the needle was left in place for 5 minutes and then slowly withdrawn and bone wax (Wax Surgical Haemostatic; REF# Z046 smi, Belgium) was used to fill the hole. The incised skin area was immediately sutured with 5.0 Ethilon™ Polyamide 6 #REF W1616T suture material. The animal was placed in a recovery cabinet (ScanBur) at approximately 35°C in a heated cabinet and provided with mashed pellet (Special Diets Services Transbreed (E) and water for a period of 30 minutes.

After a 6- or 24-hours post-surgery, animals were euthanized by CO_2_ and cervical dislocation. Blood samples was drawn from the heart into ethylenediaminetetraacetic acid (EDTA) tubes or microtubes for plasma and serum respectively, both wrapped in aluminium foil to prevent the exposure of the blood ROL to light (because retinoids are light sensitive). The brain and other organs were rapidly dissected and frozen on dry ice and stored at −70°C for quantitative polymerase chain reaction (qPCR), western blotting, and high-performance liquid chromatography (HPLC) analysis.

#### Viral construct design

The adeno-associated virus (AAV) construct (AAV9-GFP-U6-m-RBP4-shRNA, Lot# 171120#32) used to knockdown *Rbp4* was designed by Vector Biolabs. The transgene (mouse RBP4 shRNA – Genbank RefSeq: NM_01125) was packaged into AAV-9 capsid along with AAV-2 ITR and a U6 promoter. A eGFP reporter/marker was used and driven by a CMV promoter. Pre-made AAV stocks were used for the scrambled constructs based on the data sheet (Vector Biolabs). The scrambled construct name: (AAV9-GFP-U6-scrmb-shRNA, Lot# 170814-171201) sequence was not provided by Vector Biolabs. The shRNA and targeting sequence is shown below.

shRNA sequence (VBLKO-105633):

5′-CCGG-TGTGGACGAGAAGGGTCATATCTCGAGATATGACCCTTCTCGTCCACA-TTTTT -3’

Targeting sequence: TGTGGACGAGAAGGGTCATAT

Hairpin loop sequence: CTCGAG

#### Hypothalamic injection of viral vectors

0.5 μL of the viral construct (AAV9-GFP-U6-m-RBP4-shRNA, 3.5 × 10^13^ GC/ml) or 0.5 μL of the scrambled construct (AAV9-GFP-U6-scrmb-shRNA, 4.6 × 10^13^ GC/ml) were injected bilaterally into the region of the arcuate nucleus of C57BL/6 female mice selected randomly. Coordinates to reach the region of the arcuate nucleus of the mouse brain were determined in line with coronal brain sections in The Mouse Brain in Stereotaxic Coordinates.^37^ Viscotears® Liquid Gel was applied to both eyes to avoid dryness during surgery and 1.0 mg/kg of Metacam® (Boehringer Ingelheim, Germany) was prepared and 0.1 mL of the final dilution was injected subcutaneously before surgery. At the start of surgery, isoflurane was regulated between 1 – 2% in oxygen and the heating pad set at 3 to prevent heat loss from the animal. Based on the set coordinates (AP – 1.43 mm, ML – ± 0.25 mm, and DV which were determined based on the needle touching the base of the mouse skull and withdrawn 0.05 mm upward before injection), two set of small holes were drilled, using a motorised Neurostar driller, bilaterally through the skull. Subsequently, the motorised Neurostar injector was calibrated, and the Stereo-drive software was programmed to inject a volume of 0.5 μL of the viral construct over a period of 5 minutes. At the completion of the first injection the needle was left in place for 5 minutes before the subsequent transfer of the needle to the second drilled hole in the skull to inject the same quantity of the viral construct. Following the completion of the surgical procedure, the drilled holes were filled with bone-wax and the incised skin was sutured with a 6.0 Ethicon® coated Vicryl™ polyglactin 910 undyed braided absorbable suture (REF# W9500T). The animal was then placed in a recovery cage (ScanBur) at 35°C in a heated cabinet and was provided with a mashed pellet (Special Diets Services Transbreed) and water for the first period of 30 minutes of post-surgery. The animals were given post-surgical care for three weeks with free access to standard chow and water respectively.

#### mRNA transcript analysis

The total RNA was extracted from tissue samples (hypothalamus and liver) weighing < 20 mg using a RNeasy Mini Kit (Cat# 74104, Qiagen) based on the manufacturer’s protocol. The RNA concentration in the sample was measured using a NanoDrop™ 2000c Spectrophotometer (Thermo Fisher Scientific). The equipment was calibrated using 2 μL of the RNase-free water used to elute the RNA. Subsequently, 2 μL of RNA sample was used to measure the RNA concentration and the ratio of absorbance at 260 nm and 280 nm was used to estimate the RNA purity. The RNA samples were then stored at −70°C until required.

#### Complementary DNA synthesis (cDNA)

Complementary DNA was synthesised by reverse transcription using the qScript™ cDNA Synthesis Kit (Cat# 95047-100, Quanta Biosciences™). 500 ng of total RNA from tissues was added to 1 μL of qScript reverse transcriptase (RT) enzyme and 4 μL of qScript reaction mix (5X) in a 0.2 mL microtube (Cat# AX-PCR-02-A, Thistle Scientific). The final reaction mixture was subsequently made-up to 20 μL with RNase-free water. In addition, a negative control of a no RT reaction tube was set-up in each respective experiment excluding the reverse transcriptase enzyme and the final volume made-up to 20 μL. Samples and reaction mixture were kept on wet ice. Next, the tubes were mixed properly and briefly centrifuged; and were assembled on the Gene AMP® PCR System 9700 Thermocycler using the following program: 1 cycle at 22°C for 5 minutes, 1 cycle at 42°C for 30 minutes, and 1 cycle at 85°C for 5 minutes. The synthesised cDNA was stored at −20°C and used subsequently for Real-Time Quantitative Polymerase Chain Reaction (RT-qPCR).

#### Quantitative polymerase chain reaction (qPCR)

qPCR was performed (using SensiMixTM SYBR® Green (Cat# QT650-05, Bioline) on a Roche LightCycler 480 (Roche Molecular Biochemical, Indianapolis, IN)^38^ using primers in (Table S1) and *Actb* was used as the internal control standard for all samples. 12 μL of reaction mix (7.5 μL of SensiMix™ SYBR No-ROX (1X) mixed with 250 nM forward and reverse primers and the final volume was made up with RNase-free water) and 3 μL of RNA sample was added to each well of a 384-well plate (Cat# 047297400, Roche). All samples were run in technical replicate (n = 3) along with standard curves prepared from a 5-fold dilution of the stock cDNA samples mixed and blanks. To assess mRNA transcript expression levels in the samples, the following program was used: 45 cycles at 95°C for 15 seconds, 60°C for 15 seconds and 72°C for 15 seconds. The melting curve was obtained by running the plate at 95°C for 5 seconds and a 58°C for 1 minute. The mRNA expression level was measured using the Advanced Relative Quantification relative to reference transcript (*Actb*) on Roche LightCycler 480 v 1.5 software.

#### SDS-PAGE and western blotting analysis

Liver samples were homogenised in 100 μL of cold 1X lysis buffer and the protein concentration quantified using a Pierce Bicinchoninic Acid (BCA) Assay Kit (Cat# 23235, ThermoFisher Scientific) based on the manufacturer’s protocol and using an Emax Precision Microplate Reader machine (Molecular Devices) with SoftMax Pro software v 5.2. A 50 μg of protein sample was loaded onto a 12% resolving polyacrylamide gel and this was run using 1X running buffer at 150 volt (V) and for 80 min. The protein was then transferred to a nitrocellulose membrane (Cat# 10600008, GE Healthcare) at 100 V for 60 minutes. Following the 60 minutes transfer, the nitrocellulose membrane was washed twice with 1X Tris-Buffered Saline 0.1% Tween (TBST) and blocked with a 5% non-fat skimmed powered milk. Subsequently, the membrane was incubated in the respective primary antibody overnight at 4°C with constant agitation. This was followed by specific horseradish peroxidase (HRP) conjugated secondary antibody incubation. The blot was next developed using the Enhanced Chemiluminescence Kit (ECL; Cat# 11546345, Fisher Scientific) and visualised using a myECL Imager (Thermo Scientific). Western blot analysis was performed^39^ with primary antibody, against either RBP4 (1:1000; ab188230, Abcam), RBP1 (1:200; Sc-271208, Santa Cruz Biotechnology), ALDH1A1 (1:2000; ab24343, Abcam) or LRAT (1:500; 12815-1-AP, Proteintech).

#### High performance liquid chromatography (HPLC)

Retinyl palmitate and ROL were extracted from blood serum, plasma, liver, lungs, and kidney samples. In the case of blood, 100 μL of serum or plasma sample was added into 2 mL Reacti-Vial™ Small Glass Reaction Vials (ThermoFisher Scientific) along with 25 μL of internal standard (retinyl acetate) and 75 μL of 100% ethanol. After a brief vortex, 4 mL of hexane was added to the samples and vortexed for 30 seconds. Subsequently, the samples were centrifuged at 3000 rpm for 3 minutes at room temperature using a MSE Blue Force 1L centrifuge. The upper phase containing the retinoids (ROL and retinyl esters) was carefully transferred using an unplugged glass Pasteur pipette (CAT# FB50253, Fisherband®) to a fresh Reacti-Vial™ Small Glass Reaction Vials containing 500 μL HPLC water and vortexed for 30 seconds followed by centrifugation. After centrifugation, the top upper phase was transferred to an amber vial with an unplugged glass Pasteur pipette and dried under a gentle stream of nitrogen using the Evap-O-Rac System (Cole-Parmer).

Once dried, the samples were immediately re-dissolved in 50 μL of mobile phase (1400 mL acetonitrile, 300 mL methanol and 300 mL dichloromethane) all HPLC gradient grade. Subsequently, the total volume of the re-dissolved samples were transferred to polypropylene tubes for injection onto the HPLC column. 20 μL of sample was injected and run through an isocratic system (single mobile phase) on column (Gemini 5um C18 110A with guard) at 1.8 mL/minutes flow rate and between 950 and 1010 psi using a 515 HPLC Pump (ThermoFisher Scientific). The mobile phase was used as blank and human serum as a quality control (QC) sample at the start of the experiment. The QC coefficient of variation (CV) of 1.7 – 1.8 nmol/mL was used to determine the consistency of each experimental run. Also, the serum and plasma CV% was set at 10 and tissue samples set at 20 and CV values (using the formula; CV = (sample mean / standard deviation) x 100) higher than the set values were excluded. Each sample was run for a period of 20 minutes. Finally, retinoids were detected using the Water 2996-Photodiode Array Detector at 325 nm absorbance. Retinyl acetate (internal standard) was detected at 2.95 minutes, ROL 2.61 minutes and retinyl palmitate 12.30 minutes respectively. The column temperature was set at 28°C and auto-sampler temperature at 8°C.

The same extraction procedure was used for tissue samples with the exception that weight was precisely measured. Approximately 100 mg of liver (or ∼500 mg of kidney or lungs) samples were weighed (SI-114, Derner Instrument) and homogenised in 2 mL of cold 1X phosphate buffer sulphate using a motorised homogeniser (200 Pro-Scientific Inc) for 15 seconds. The homogenate was left on wet-ice constantly and in a room with low light to avoid enzymatic oxidation of the retinyl esters. Subsequently, 200 μL of the homogenate was transferred to a glass Reacti-Vial™ Small Glass Reaction Vials and 100 μL of internal standard and 100 μL of 10% butylated hydroxytoluene in 100% ethanol was added (1:1; v/v). The mixture was briefly vortexed, and 4 mL of hexane was added to the samples and was vortexed for 30 seconds. At this stage, the same procedure used to extract retinoids from serum or plasma was followed as described above.

### Quantification and statistical analysis

All data were analysed by a two-tailed Student’s *t* test using the IBM SPSS Statistic v 25 program and results were expressed as Means ± SEM. Subsequently, values of p < 0.05 were considered significant. Results were presented using GraphPad Prism (v 9.4.1).

## Data Availability

• All data produced in this study are included in the published article and its supplemental information or are available from the lead contact upon request. • This paper does not report original code. • Any additional information required to reanalyse the data reported in this paper is available from the lead contact upon request.

## Supplemental information

Table S1. Genes and primer sequence for qPCR experiment, related to STAR Methods
