# Supplementary material for: Control by the brain of vitamin A homeostasis
Source: iScience. 2023 Jul 13;26(8):107373. doi: 10.1016/j.isci.2023.107373 (PMC10432198; doi:10.1016/j.isci.2023.107373)
Supplement: Table S1. Genes and primer sequence for qPCR experiment, related to STAR Methods [file mmc1.pdf]

iScience, Volume *26*

Supplemental information

Control by the brain of vitamin A homeostasis

Peter I. Imoesi, Cristian M. Olarte-Sánchez, Lorenzo Croce, William S. Blaner, Peter J. Morgan, Lora Heisler, and Peter McCaffery

Supplementary Table 1: Genes and primer sequence for qPCR experiment, related to STAR Method.

Gene	Forward Primer Sequence	Reverse Primer Sequence
Aldh1a1(NM_022407.3)	CTCACTGCTCTTCACCATGGC	TCCATGTGAGAGGAGATCGC
Lrat (NM_022280.2)	TGGGCTGACCCCCTACAGC	TCTCGTGAAACTTCTCAGCCTGCGG
Rbp1 (NM_012733.3)	AGCGCATTGGCAGCCACAGT	AGGCCACGTTGACATCGAGCG
Rarb (NM_011243.1)	ACACCACGAATTCCAGCGCTGAC	TACAGCCCGGGGAGCATCGT
Rbp4 (NM_013162.1)	TTCTGATTAGCTCTCATCCA	TGGACGATGGTTTTAATGTC
Actb (NM_007393.3)	CCACACCCGCCACCAGTTCG	TACAGCCCGGGGAGCATCGT
